# Bicarbonate and Ca^2+^ Sensing Modulators Activate Photoreceptor ROS-GC1 Synergistically

**DOI:** 10.3389/fnmol.2016.00005

**Published:** 2016-01-28

**Authors:** Teresa Duda, Alexandre Pertzev, Clint L. Makino, Rameshwar K. Sharma

**Affiliations:** ^1^Research Divisions of Biochemistry and Molecular Biology, The Unit of Regulatory and Molecular Biology, Salus UniversityElkins Park, PA, USA; ^2^Department of Ophthalmology, Massachusetts Eye and Ear Infirmary and Harvard Medical SchoolBoston, MA, USA

**Keywords:** bicarbonate, calcium-binding protein, guanylate cyclase (guanylyl cyclase), guanylate cyclase activating protein (GCAP), phototransduction, S100 proteins, signal transduction, second messenger

## Abstract

Photoreceptor ROS-GC1, a prototype subfamily member of the membrane guanylate cyclase family, is a central component of phototransduction. It is a single transmembrane-spanning protein, composed of modular blocks. In rods, guanylate cyclase activating proteins (GCAPs) 1 and 2 bind to its juxtamembrane domain (JMD) and the C-terminal extension, respectively, to accelerate cyclic GMP synthesis when Ca^2+^ levels are low. In cones, the additional expression of the Ca^2+^-dependent guanylate cyclase activating protein (CD-GCAP) S100B which binds to its C-terminal extension, supports acceleration of cyclic GMP synthesis at high Ca^2+^ levels. Independent of Ca^2+^, ROS-GC1 activity is also stimulated directly by bicarbonate binding to the core catalytic domain (CCD). Several enticing molecular features of this transduction system are revealed in the present study. In combination, bicarbonate and Ca^2+^-dependent modulators raised maximal ROS-GC activity to levels that exceeded the sum of their individual effects. The F^514^S mutation in ROS-GC1 that causes blindness in type 1 Leber’s congenital amaurosis (LCA) severely reduced basal ROS-GC1 activity. GCAP2 and S100B Ca^2+^ signaling modes remained functional, while the GCAP1-modulated mode was diminished. Bicarbonate nearly restored basal activity as well as GCAP2- and S100B-stimulated activities of the F^514^S mutant to normal levels but could not resurrect GCAP1 stimulation. We conclude that GCAP1 and GCAP2 forge distinct pathways through domain-specific modules of ROS-GC1 whereas the S100B and GCAP2 pathways may overlap. The synergistic interlinking of bicarbonate to GCAPs- and S100B-modulated pathways intensifies and tunes the dependence of cyclic GMP synthesis on intracellular Ca^2+^. Our study challenges the recently proposed GCAP1 and GCAP2 “overlapping” phototransduction model (Peshenko et al., 2015b).

## Introduction

ROS-GCs are Ca^2+^-modulated members of the membrane guanylate cyclase family (reviewed in Sharma and Duda, 2014). Originally discovered in retina where they play an integral role in phototransduction in the outer segments of photoreceptors (Margulis et al., 1993; Goraczniak et al., 1994; Duda et al., 1996), ROS-GCs are also present in the photoreceptor cell bodies (Rambotti et al., 1999), photoreceptor synaptic region (Duda et al., 2002; Venkataraman et al., 2003), Muller cells (Rambotti et al., 1999) and in ganglion cells (Krishnan et al., 2004). Outside the retina, they are expressed in pinealocytes (Venkataraman et al., 2000), in olfactory bulb neurons (Duda et al., 2001), in the anterior portion of gustatory epithelium (Duda and Sharma, 2004) and in sperm (Jankowska et al., 2014). Thus, ROS-GCs direct multimodal cellular signal transductions (Sharma et al., 2015).

In outer segments of retinal rods and cones, cyclic GMP generated by ROS-GCs serves as the second messenger of the phototransduction cascade. In darkness, cytoplasmic cyclic GMP keeps a fraction of the cyclic nucleotide-gated (CNG) channels open, through which Na^+^ and some Ca^2+^ enter the photoreceptor (Pugh et al., 1997; Imanishi et al., 2004; Sharma, 2010; Sharma and Duda, 2014). Upon photon absorption, a rhodopsin molecule catalyzes the exchange of GTP for GDP bound to the G protein, transducin. With GTP-bound, transducin activates a phosphodiesterase (PDE) which then rapidly hydrolyzes cyclic GMP, closing CNG channels. The ensuing membrane hyperpolarization spreads across the inner segment to the synaptic terminal, where it alters synaptic transmission. To return to the resting state, photoexcited rhodopsin is quenched by phosphorylation and arrestin binding. After hydrolysis of GTP, transducin loses the ability to activate PDE. Cyclic GMP levels are restored by ROS-GCs. The light-induced closure of the CNG channels results in a fall in [Ca^2+^]_i_. One or more guanylate cyclase activating proteins (GCAPs) respond to the fall in [Ca^2+^]_i_ and stimulate ROS-GCs to synthesize cyclic GMP at a much faster rate. This negative feedback loop limits the size of the single photon response and hastens its recovery. Some cones express an additional Ca^2+^ sensor, S100B, that stimulates ROS-GC activity at high levels of Ca^2+^ (Wen et al., 2012). Recently, another intriguing feature of ROS-GC linked phototransduction has been observed. Through a unique Ca^2+^-independent mechanism, ROS-GC catalytic activity is stimulated by bicarbonate to provide for an increase in circulating current, quickened flash responses, and reduced relative sensitivity (Duda et al., 2015). These bicarbonate effects may for the first time explain the observed larger photoreceptor response in the ERG of the isolated retina treated with bicarbonate (Donner et al., 1990; Koskelainen et al., 1993) or the reduced photoreceptor response in the ERGs of human subjects and animal models treated with carbonic anhydrase inhibitors (Broeders et al., 1988; Odom et al., 1994; Findl et al., 1995).

**Figure 1:**
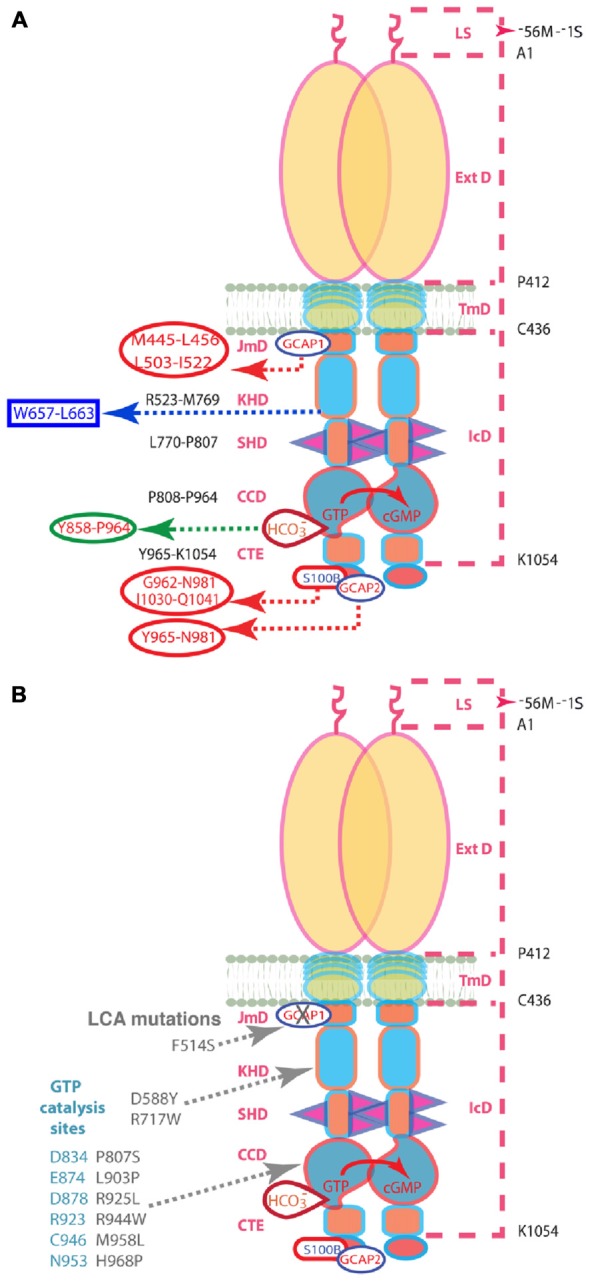
**(A)** Schematic representation of the structural domains of the ROS-GC1 homodimer. The dashed lines on the right outline the boundaries of its segments. LS, leader sequence that is absent in the mature form of the ROS-GC1 expressed in outer segments; ExtD, extracellular domain; TmD, transmembrane domain; IcD, intracellular domain. All functional signaling subdomains are housed in IcD; their designated names and the amino acids residues constituting their boundaries are indicated on the left. The binding sites for GCAP1, GCAP2, S100B, and bicarbonate are shown for one monomer but the ROS-GC1 dimer binds two GCAP1, two GCAP2, or two S100B subunits and is likely capable of binding at least two bicarbonate molecules irrespective of which Ca^2+^-sensing subunits are attached. The figure has been modified from Duda et al. (2015). The GCAP1 signal originates at the L^503^-I^522^ site in JMD and migrates downstream through the successive kinase homology domain (KHD) and signaling helix domain (SHD) to reach core catalytic domain (CCD), a central location for the translation of all the signals into cyclic GMP synthesis. Signals generated by GCAP2 and S100B originate from CTE (C-terminal extension) and migrate upstream to CCD to be translated. The ^657^WTAPELL^663^ motif is critical for the signaling of both GCAPs, however, it has no role in controlling the basal catalytic activity of the cyclase or in the binding of the GCAPs. In contrast to GCAP2 signaling, SHD is obligatory for GCAP1 signaling. Bicarbonate signals CCD activation independent of [Ca^2+^]. All other signaling mechanisms are sensitive to [Ca^2+^]. For clarity, the depicted domains are not proportional to the scale. **(B)** Mutations in ROS-GC1 that cause LCA (gray). The F^514^S mutation residing in the GCAP1-modulated region of the JMD disabled the basal catalytic activity and compromised regulation by GCAP1 but left the regulatory activities of the GCAP2, S100B, and bicarbonate intact. The D^588^Y and R^717^W mutations in the KHD reduce basal activity and prevent GCAP1 binding (Peshenko et al., 2010). The P^807^S and L^903^P mutations in the CCD lower basal activity and GCAPs-stimulated activities (Tucker et al., 2004). The other CCD mutations that cause LCA are thought to eradicate catalytis cativity (Rozet et al., 2001). The steric arrangements of the D^834^, E^874^, D^878^, R^923^, C^946^, and N^953^ residues of the CCD (blue-gray) are predicted to be negatively influenced by the LCA1-linked mutations. Note: the amino acid residue numbers reflect their positions in the mature bovine protein (Goraczniak et al., 1994).

In the present study, molecular biological, genetic and biochemical approaches are used to define the interplay between Ca^2+^-dependent mechanisms and bicarbonate on photoreceptor ROS-GC1 activity. ROS-GC1 is a single transmembrane-spanning protein, subdivided into modular blocks (Duda et al., 2015; Figure 1A). GCAP1 binds to an intracellular, juxtamembrane domain (JMD) whereas GCAP2 and S100B bind to sites on the carboxy terminal extension. Bicarbonate binds to the core catalytic domain (CCD) located between the binding sites for the Ca^2+^-sensing subunits (Duda and Sharma, 2010; Duda et al., 2015). An F→S substitution in the JMD of ROS-GC1 is responsible for Leber’s congenital amaurosis (LCA) type 1, a blinding retinal disease. It severely impairs the intrinsic activity of the enzyme and suppresses modulation by GCAP1 (Duda et al., 1999). This mutant was used to probe the intramolecular pathways for controlling ROS-GC1 activity (Figure 1B).

## Materials and Methods

### Measurement of Recombinant ROS-GC1 Activity

COS7 cells were transfected with cDNA encoding bovine ROS-GC1 or its F^514^S mutant (Duda et al., 1999) by the calcium co-precipitation technique (Sambrook et al., 1989). Sixty-four hours post-transfection, the cells were washed with 10 mM Mg^2+^ in 50 mM Tris-HCl buffer pH 7.4, homogenized, and the particulate fraction was pelleted by centrifugation.

Membrane samples were individually incubated with sodium bicarbonate in the absence or presence of recombinant bovine GCAP1 or GCAP2 purified as described in Duda et al. (2011) or S100B (Sigma). The assay mixture (25 μl) consisted of (mM): 10 theophylline, 15 phosphocreatine, 50 Tris-HCl; pH 7.5, and 20 μg creatine kinase (Sigma). Unless otherwise stated, for experiments with GCAPs, 1 mM EGTA was present in the reaction mixture and for experiments with S100B, 1 μM Ca^2+^ was present. Ca^2+^ concentration in assay mixture was calculated using Ca^2+^ calculator^1^. The reaction was initiated by addition of the substrate solution (4 mM MgCl_2_ and 1 mM GTP, final concentrations) and incubated at 37°C for 10 min. The reaction was terminated by the addition of 225 μl of 50 mM sodium acetate buffer, pH 6.2, followed by heating on a boiling water bath for 3 min. The amount of cyclic GMP formed was determined by radioimmunoassay (Paul et al., 1987).

### Preparation of Native ROS-GC from Mouse

Care of the experimental animals conformed to the protocols approved by the IACUC at Salus University and was in strict compliance with NIH guidelines. Rod outer segments were isolated from the retinas of GCAPs^−/−^ (Mendez et al., 2001; Pertzev et al., 2010) and S100B^−/−^ (Wen et al., 2012) mice and their littermate controls (WT) according to the original protocol of Nickell et al. (2007). Outer segment membranes of GCAPs^−/−^ mice and their WT controls were prepared in the presence of 1 mM EGTA and of the S100B^−/−^ mice and their WT controls in the presence of 10 μM Ca^2+^. These membranes were assayed for guanylate cyclase activity using methods identical to those for COS cell membranes, except that 10 μM zaprinast was added to inhibit intrinsic PDE6 activity.

## Results

### Recovery of the Deficit in Catalytic Activity of the F^514^S Mutant ROS-GC1 with Bicarbonate

**Figure 2:**
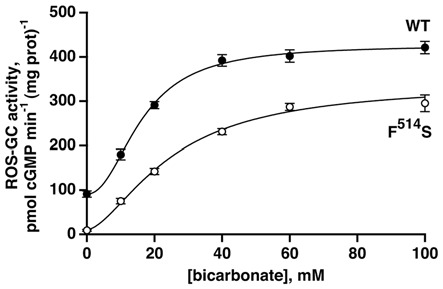
**Activation of recombinant WT and mutant F^514^S ROS-GC1 by bicarbonate.** The experiments were done in triplicate and repeated five times; the 100 mM bicarbonate concentration was only included in two experiments. Collected results were fit with a Hill function: activity = basal activity + (maximal activity − basal activity)([bicarbonate]^n^/([bicarbonate]^n^ + ED_50_^n^)), but symbols plot mean ± standard deviation for clarity. For the WT ROS-GC1, the Hill coefficient was 2.2 and the ED_50_ was 16 mM while for the F^514^S mutant, the Hill coefficient was 1.6 and the ED_50_ was 25 mM.

The F^514^S substitution in bovine ROS-GC1 corresponds to the naturally occurring F^565^S mutation in human retinal GC1 that causes LCA type 1 (Perrault et al., 1996; Figure 1B). It cripples the ability of the cyclase to synthesize cyclic GMP (Duda et al., 1999; Rozet et al., 2001). As a first step in dissecting the signaling pathways within ROS-GC1 for bicarbonate, GCAPs and S100B, recombinant F^514^S ROS-GC1 was tested for basal and bicarbonate-dependent activities. As expected (Duda et al., 1999), the basal activity of the mutant, 11 pmol cyclic GMP min^−1^ (mg prot)^−1^, was far lower than the activity of WT ROS-GC1, 91 pmol cyclic GMP min^−1^ (mg prot)^−1^. When challenged with rising concentrations of bicarbonate, both cyclases responded with an increase in activity (Figure 2). WT ROS-GC1 was stimulated 4.7-fold by bicarbonate with a Hill coefficient of 2.2. The ED_50_ of 16 mM was somewhat lower than was observed previously (Duda et al., 2015). The F^514^S mutant was stimulated by more than 30-fold with half-maximal stimulation at 25 mM bicarbonate although the F^514^S mutation introduced a constant decrement across all bicarbonate concentrations tested. That implies that the two factors (F^514^S mutation and bicarbonate) operate independently on the catalytic domain of ROS-GC1.

### Disrupted Regulation by GCAP1 But Not by GCAP2 in the F^514^S Mutant

**Figure 3:**
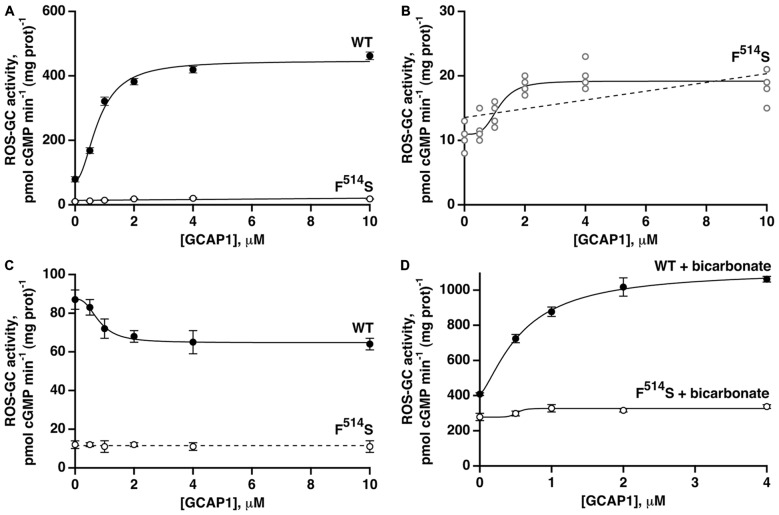
**Residual effects of GCAP1 on the activity of recombinant F^514^S mutant ROS-GC1.** Symbols with error bars plot mean ± standard deviation, but traces show fits of collected results to a Hill function (continuous lines). **(A)** Stimulation of ROS-GC1 and its F^514^S mutant by GCAP1 in the presence of 1 mM EGTA (nominally 10 nM Ca^2+^). For WT ROS-GC1, the change in activity was 5.7-fold, the Hill coefficient was 2.0 and the ED_50_ was 0.8 μM. For the mutant, the change in activity was approximately 1.7-fold. Experiments were done in triplicate and repeated three times for WT and four times for the mutant. **(B)** Collected results for F^514^S ROS-GC1 from **(A)** plotted on a rescaled ordinate. Linear regression (dashed line) yielded a slope that differed significantly from zero, *p* < 0.003. **(C)** GCAP1 inhibition of ROS-GC1 but not of the F^514^S mutant at high, 2 μM Ca^2+^. Continuous trace shows a fit of the WT ROS-GC1 scatter plot with a Hill function: activity = (maximal activity − minimal activity)/(1 + ([GCAP1]/ED_50_)^n^) + minimal activity. Activity decreased by 1.3-fold, the Hill coefficient was 2.7, and the ED_50_ was 0.8 μM. The slope obtained from a linear regression analysis of F^514^S results was not significant, so the dashed trace shows a horizontal line with an intercept of 11.5, the mean of all F^514^S values. Experiments were done in triplicate and repeated twice. **(D)** Potentiated GCAP1 activation of ROS-GC1 but not the F^514^S mutant by bicarbonate. In the presence of 1 mM EGTA, 50 mM bicarbonate and indicated concentrations of GCAP1, activity of the WT ROS-GC1 climbed 2.7-fold whereas that of the F^514^S mutant increased ~1.2-fold. For the WT ROS-GC1, the Hill coefficient was 1.4 and the ED_50_ was 0.6 μM. The experiments were done in triplicate and repeated three times.

With the addition of GCAP1 at low [Ca^2+^], ROS-GC1 responded with a dose-dependent increase of cyclic GMP synthesis, a phenomenon well established for recombinant ROS-GC1 expressed in heterologous systems (Duda et al., 1996) and for ROS-GC1 present in native, photoreceptor outer segment membranes (Howes et al., 2002; Pennesi et al., 2003). In the present study, the maximal ROS-GC1 activity achieved with GCAP1 was 460 pmol cyclic GMP min^−1^ (mg prot)^−1^ (Figure 3A). At high [Ca^2+^], GCAP1 inhibited WT ROS-GC1 activity in a dose-dependent fashion (Figure 3C).

When the F^514^S mutant was tested under the same experimental conditions, increasing concentrations of GCAP1 stimulated slightly, its activity at low Ca^2+^ concentration (Figure 3A). The slope obtained from a linear regression of the mutant’s activity against [GCAP1] differed significantly from zero (Figure 3B). A small trend that did not reach significance was noted in an earlier study (Duda et al., 1999). At high [Ca^2+^], GCAP1 had no measurable effect on the mutant’s activity, contrary to the reductive effect that it had on WT ROS-GC1 activity (Figure 3C).

Fifty millimolar bicarbonate enhanced GCAP1 mediated stimulation of WT ROS-GC1 activity, lowered the ED_50_ value for GCAP1 (0.6 μM with and 0.8 μM without bicarbonate, respectively) and raised V_max_ to ~1110 pmol cyclic GMP min^−1^ (mg prot)^−1^ (Figure 3D). This level surpassed 780 pmol cyclic GMP min^−1^ (mg prot)^−1^, found by adding the effect of bicarbonate to the GCAP1 V_max_. Bicarbonate by itself stimulated the mutant’s activity from 11 to 279 pmol cyclic GMP min^−1^ (mg prot)^−1^, and may have resulted in some additional increase in guanylate cyclase activity over the range of GCAP1 concentrations tested (Figure 3D). Linear regression analysis returned a positive slope that differed from zero with *p* < 0.005 (not shown). It is not yet clear whether bicarbonate and GCAP1 at low Ca^2+^ operated synergistically on the mutant ROS-GC1 activity.

**Figure 4:**
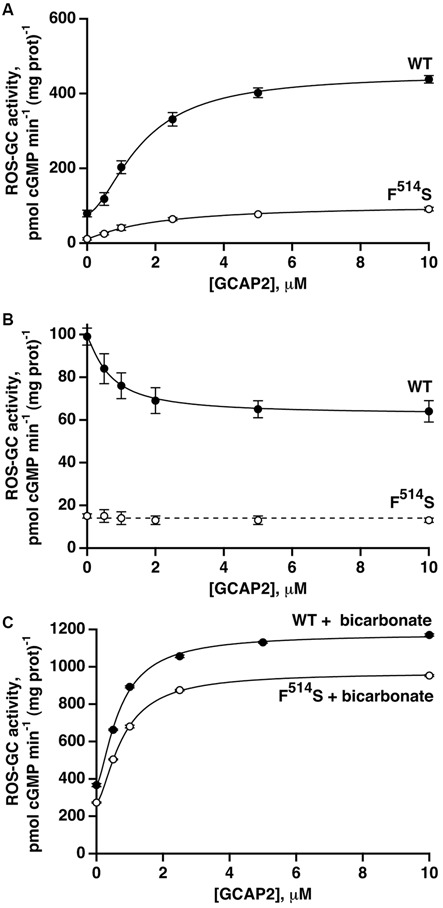
**GCAP2 effect on the activity of recombinant F^514^S mutant of ROS-GC1. (A)** GCAP2-stimulated activities of ROS-GC1 and its F^514^S mutant at low Ca^2+^. V_max_ for WT ROS-GC1 was 5.7-fold greater than the basal activity, the Hill coefficient was 1.6 and the ED_50_ was 1.6 μM. For the mutant, activity increased 9.3-fold, the Hill coefficient was 1.1 and the ED_50_ was 1.9 μM. **(B)** GCAP2 inhibition of ROS-GC1 but not the F^514^S mutant at high, 2 μM Ca^2+^. For WT ROS-GC1, the decrease in activity was 1.6-fold, Hill coefficient was 1.2 and ED_50_ was 0.7 μM. The slope from a linear regression of the results for F^514^S was not significant. The dashed line has a slope of zero and an intercept of 14 pmol cGMP min^−1^ (mg prot)^−1^ which was the mean of all F^514^S values. **(C)** Bicarbonate potentiated GCAP2 activation of ROS-GC1 and of the F^514^S mutant. With 50 mM bicarbonate, 1 mM EGTA and increasing concentrations of GCAP2, WT ROS-GC1 activity increased 3.2-fold with a Hill coefficient of 1.6 and an ED_50_ of 0.7 μM, whereas for F^514^S ROS-GC1, activity increased 3.5-fold with a Hill coefficient of 1.6 and ED_50_ of 0.8 μM. The experiments were done in triplicate and repeated three times.

A quantitatively different picture emerged when GCAP2-dependent activity was analyzed. At low Ca^2+^, the activity of WT ROS-GC1 increased about 5.7-fold in the presence of GCAP2 (Figure 4A). The activity exhibited by the F^514^S mutant rose about 9.3-fold, nevertheless, the maximal activity of 91 pmol cyclic GMP min^−1^ (mg prot)^−1^ remained considerably lower than the maximum of 439 pmol cyclic GMP min^−1^ (mg prot)^−1^ for WT ROS-GC (Figure 4A). The ED_50_ value of 1.9 μM for GCAP2 of the mutant ROS-GC1 was comparable to the value of 1.6 μM for the WT ROS-GC1. At high Ca^2+^, GCAP2 inhibited in a dose-dependent fashion the activity of ROS-GC1 but not that of its F^514^S mutant (Figure 4B).

The intensified response of the F^514^S mutant to GCAP2 at low Ca^2+^ contrasted with the attenuated response to GCAP1 and supported the notion that each GCAP targets separate binding domains on ROS-GC1 and employs different signaling pathways in modulating ROS-GC1 activity (Duda et al., 1996, 2012; Krishnan et al., 1998; Lange et al., 1999; Koch et al., 2010; Koch and Dell’Orco, 2013). Our results do not support the recent proposition that ROS-GC binding sites for GCAP1 and GCAP2 overlap (Peshenko et al., 2015a,b), which was deduced through indirect studies using total mouse retina as a source of the ROS-GC1. Previous studies have established that the retina, besides ROS-GC1 and ROS-GC2, contains other membrane guanylate cyclases, e.g., C-type natriuretic peptide receptor guanylate cyclase (Duda et al., 1993), and atrial natriuretic factor (ANF) receptor guanylate cyclase (ANF-RGC; Pardhasaradhi et al., 1994) and also that the ROS-GC1 present in photoreceptor synapses and in ganglion cells is complexed respectively with S100B and neurocalcin δ (Venkataraman et al., 2003; Krishnan et al., 2004).

With bicarbonate present, the effects of GCAP2 at low Ca^2+^ on WT and mutant ROS-GC1 activities were each enhanced (Figure 4C). With the WT, the V_max_ of ~1150 pmol cyclic GMP min^−1^ (mg prot)^−1^ exceeded the summed effects of bicarbonate and GCAP2, ~720 pmol cyclic GMP min^−1^ (mg prot)^−1^, and the ED_50_ for GCAP2 was lowered from 1.6 to 0.7 μM. With the F^514^S mutant, V_max_ reached ~970 pmol cyclic GMP min^−1^ (mg prot)^−1^, very nearly as high as that of WT ROS-GC1 and several-fold higher than expected from adding the effects of bicarbonate and GCAP2, 360 pmol cyclic GMP min^−1^ (mg prot)^−1^. As with the WT ROS-GC1, 50 mM bicarbonate lowered the ED_50_ of the F^514^S mutant for GCAP2 by about 2-fold. Hence, bicarbonate nearly recouped the full deficit imparted by the F^514^S mutation in the presence of GCAP2 at low Ca^2+^.

### Synergic Modulation of ROS-GC1 Activity by Bicarbonate and S100B

**Figure 5:**
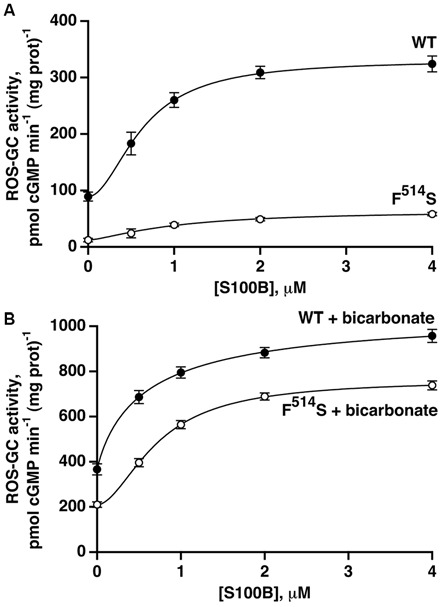
**Retention of S100B activation by the F^514^S mutant in the absence of (A) and in the presence of (B) bicarbonate.** One micromolar Ca^2+^ was present in the assay mixture. The experiments were done in triplicate and repeated three times. In the absence of bicarbonate, activation by S100B was 3.7-fold for WT ROS-GC1 with a Hill coefficient of 1.9 and an ED_50_ of 0.6 μM, whereas for the mutant, activation was 5.7-fold with a Hill coefficient of 1.5 and an ED_50_ of 1.0 μM. With the addition of 50 mM bicarbonate, activation of WT ROS-GC1 by S100B was 2.9-fold, the Hill coefficient was 0.9 and ED_50_ was 0.6 μM. For the mutant, activation was 3.6-fold, the Hill coefficient was 1.8 and ED_50_ was 0.7 μM.

The second limb of the Ca^2+^-modulated ROS-GC1 activity, functional in cone outer segments (Duda et al., 2015), involves stimulation by S100B protein at Ca^2+^ concentrations ≥400 nM. To analyze the relationship between S100B- and bicarbonate-dependent ROS-GC1 activations, recombinant ROS-GC1 and its F^514^S mutant were assayed for cyclic GMP synthesis in the presence and absence of S100B and/or bicarbonate. S100B alone stimulated both cyclases in a dose-dependent fashion (Figure 5A) with an ED_50_ for S100B that was slightly higher for the F^514^S mutant than for the WT ROS-GC1, 1.0 and 0.6 μM, respectively. Although both cyclases were activated by S100B, the maximal activity of 60 pmol cyclic GMP min^−1^ (mg prot)^−1^ achieved by the mutant remained depressed from that of WT ROS-GC1, 330 pmol cyclic GMP min^−1^ (mg prot)^−1^.

Fifty millimolar bicarbonate boosted WT ROS-GC1 activity at all concentrations of S100B (Figure 5B). The maximal achieved activity of 1070 pmol cyclic GMP min^−1^ (mg prot)^−1^ exceeded the additive value of 580 pmol cyclic GMP min^−1^ (mg prot)^−1^, indicating that as was the case with GCAPs (Duda et al., 2015), bicarbonate and S100B acted synergetically. The effect of bicarbonate on the response of the F^514^S mutant to S100B was even more compelling. The mutant synthesized cyclic GMP with a V_max_ of 760 pmol cyclic GMP min^−1^ (mg prot)^−1^, a rate almost as high as for WT ROS-GC1. Furthermore, this V_max_ was 2.9-fold greater than the value of 260 pmol cyclic GMP min^−1^ (mg prot)^−1^ predicted from summing the effects of bicarbonate and S100B. Thus, bicarbonate significantly restored the F^514^S mutant catalytic activity and responsiveness to both low and high Ca^2+^-dependent modulators, GCAP2 and S100B, but failed to do so for GCAP1.

### Synergy of Bicarbonate and Ca^2+^-dependent Modulators of ROS-GC Activity in Native Membranes

**Figure 6:**
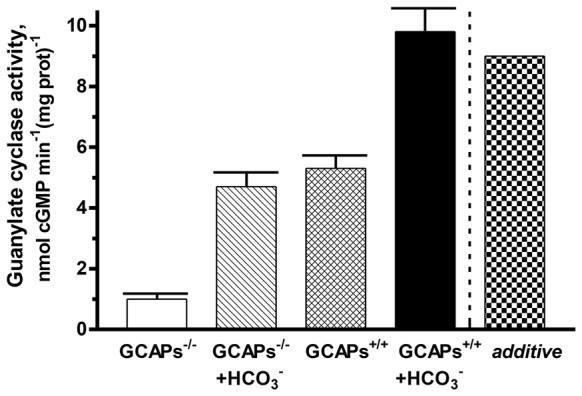
**Synergistic regulation of native ROS-GC activity by GCAPs and bicarbonate.** Outer segment membranes of GCAPs^−/−^ and GCAPs^+/+^ mice were assayed for guanylate cyclase activity in the presence of 1 mM EGTA with or without 50 mM bicarbonate. The column labeled “*additive*” plots the GCAPs^+/+^ activity at low Ca^2+^ plus the GCAPs^−/−^ with bicarbonate activity minus the basal GCAPs^−/−^ activity. Error bars show standard deviations. The experiment was done in triplicate and repeated two times.

To determine the interplay between GCAPs and bicarbonate in regulating native ROS-GC activity, membranes of outer segments from the retinas of GCAP1, 2 KO mice (GCAPs^−/−^) and their littermate controls (GCAPs^+/+^) were assayed for guanylate cyclase activity at low Ca^2+^ with or without 50 mM bicarbonate (Figure 6). The specific cyclase activity in the membranes isolated from the GCAPs^−/−^ outer segments was 1 ± 0.1 nmol cyclic GMP min^−1^ (mg prot)^−1^ and it was 5.3 ± 0.4 nmol cyclic GMP min^−1^ (mg prot)^−1^ in the membranes isolated from the controls. The difference in the activities reflects the stimulatory effect of GCAPs on the cyclase activity at low Ca^2+^. Upon addition of 50 mM bicarbonate, activity rose to 4.7 ± 0.4 nmol cyclic GMP min^−1^ (mg prot)^−1^ in the GCAPs^−/−^ membranes and to 9.8 ± 0.8 nmol cyclic GMP min^−1^ (mg prot)^−1^ in the GCAPs^+/+^ membranes. Thus, the combined effects of GCAPs and bicarbonate on ROS-GC activity were synergic. Had they been additive cyclase activity would have been 4.7 *+* (5.3 − 1) = 9 nmol cyclic GMP min^−1^ (mg prot)^−1^ (Figure 6: last column, “*additive*”). It was interesting to note that the absolute activities of ROS-GC from native murine membranes with and without GCAPs were quite a bit higher than those of recombinant bovine ROS-GC under the same conditions and that the synergic effect observed with native membranes was not as prominent as observed in the recombinant system (Duda et al., 2015), nonetheless, the phenomenon was robust. The differences between the two systems may have been related to the experimental conditions and to the unknown stoichiometry between cyclase and GCAPs in native membrane preparations whereas in the recombinant system ROS-GC1 was exposed individually to strictly controlled concentrations of GCAP1 or GCAP2 and bicarbonate (Duda et al., 2015).

**Figure 7:**
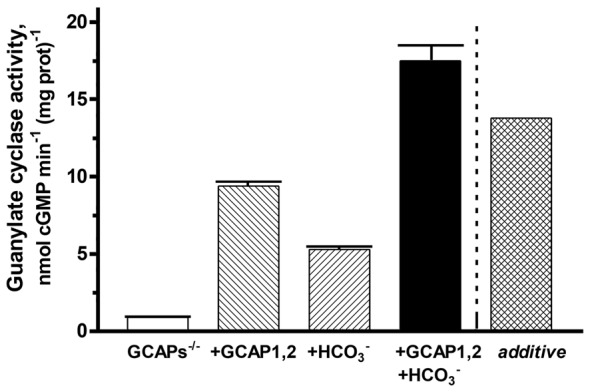
**Synergy of GCAPs and bicarbonate in stimulating ROS-GC1 activity determined through reconstitution.** Guanylate cyclase activity of GCAPs^−/−^ mouse outer segment membranes increased after reconstitution with 4 μM each of GCAP1 and GCAP2. The addition of 50 mM raised activity in the presence of GCAPs to a level that was higher than predicted from summing the individual effects of GCAPs and bicarbonate (“*additive*”). One mM EGTA was present in the assay mixture. The experiment was done in triplicate and repeated two times.

To verify the synergy between GCAPs and bicarbonate in modulating ROS-GC1 activity, a reconstitution experiment was performed on the membranes of GCAPs^−/−^ mouse outer segment membranes (Figure 7). Simultaneous addition of GCAP1 and GCAP2 at equimolar, 4 μM concentrations resulted in stimulation of ROS-GC1 activity up to 9.4 nmol cyclic GMP min^−1^ (mg prot)^−1^, an increase of 8.5 nmol cyclic GMP min^−1^ (mg prot)^−1^. Fifty mM bicarbonate increased the activity up to 5.3 nmol cyclic GMP min^−1^ (mg prot)^−1^. Additivity of GCAPs and bicarbonate effects should have yielded an activity of 13.8 nmol cyclic GMP min^−1^ (mg prot)^−1^. Instead, activity rose to 17.5 nmol cyclic GMP min^−1^ (mg prot)^−1^ demonstrating again that both GCAPs and bicarbonate are synergic modulators of ROS-GC1 activity.

**Figure 8:**
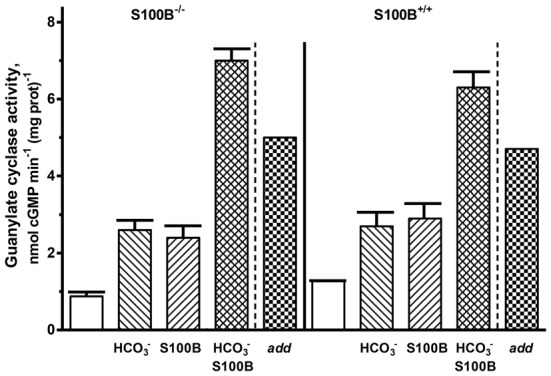
**Synergistic regulation of ROS-GC activity in the membranes of murine outer segments by S100B and bicarbonate.** The guanylate cyclase activity of outer segment membranes from S100B^−/−^ and S100B^+/+^ control mice with a combination of 2 μM S100B and 50 mM bicarbonate in the presence of 1 μM Ca^2+^ surpassed the sum (“*add*”) of the activities obtained with S100B and bicarbonate applied separately. The experiment was done in triplicate and repeated two times.

For confirmation of the interplay between bicarbonate and S100B in native ROS-GC1 signaling, outer segment membranes from the retinas of the S100B-KO mice (S100B^−/−^) and their littermate controls (S100B^+/+^) were assayed for guanylate cyclase activity (Figure 8). Compared to the specific cyclase activity in control mouse outer segment membranes, that for S100B^−/−^ mouse membranes was slightly lower, reflecting the S100B stimulatory effect on cyclase activity (Figure 8). Although the magnitude of the difference was small, it is likely to be physiologically significant because S100B is expressed only in cone outer segments (Wen et al., 2012) and cones comprise only about 3% of the photoreceptor population in the murine retina (Carter-Dawson and LaVail, 1979).

Both types of membranes responded to 50 mM bicarbonate with increased cyclase activity. The activity rose from 0.9 up to 2.6 nmol cyclic GMP min^−1^ (mg prot)^−1^ in the S100B^−/−^ membranes and from 1.3 to 2.7 nmol cyclic GMP min^−1^ (mg prot)^−1^ in the S100B^+/+^ membranes. Exposing both types of membranes to 2 μM exogenous S100B resulted in the cyclase activation of 2.4 and 2.9 nmol cyclic GMP min^−1^ (mg prot)^−1^ for the S100B^−/−^ and S100B^+/+^, respectively. When the membranes were contemporaneously treated with 2 μM S100B and 50 mM bicarbonate, their combined effects raised ROS-GC activity to 6–7 nmol cyclic GMP min^−1^ (mg prot)^−1^. Had the effects of S100B and bicarbonate been additive, the rate of cyclic GMP formed would have been no higher than 5 nmol cyclic GMP min^−1^ (mg prot)^−1^. These results demonstrate that in native membranes bicarbonate was able to activate ROS-GC alone and in concert with GCAP1, GCAP2 or S100B. When acting together, similar to the recombinant system, there was synergy between these Ca^2+^ sensors and bicarbonate in modulating ROS-GC activity.

## Discussion

GCAPs, S100B and bicarbonate interact with ROS-GC1 through distinctly different switching modes. At the sub-molecular level the functional membrane guanylate cyclase is a homodimer formed by three contact regions of the two subunits (Figure 1A). Based on the analysis of ANF-RGC (Ogawa et al., 2009), there is head-to-head contact at the extracellular domain (ExtD). The signaling helix domain (SHD) forms a second contact region (Duda et al., 2012), while the third contact is made at the CCD (Venkataraman et al., 2008), where two monomers assume an antiparallel conformation (Duda et al., 2012). Ca^2+^ sensitivity is conferred to the ROS-GC1 dimer by the binding of a pair of GCAP1, GCAP2 or S100B subunits. Remarkably, the signal generated by GCAP1 originates from the opposite side of the CCD than those generated by GCAP2 and S100B.

The GCAP1-modulated signal involves its myristoyl group (Hwang and Koch, 2002a,b; Hwang et al., 2003) and initiates at a binding site that includes L^503^-I^522^ region of the intracellular JMD (Lange et al., 1999). Two residues in the KHD may also participate because D^639^Y and R^768^W mutations in human ROS-GC1 preclude GCAP1 binding and cause LCA (Peshenko et al., 2010). These residues correspond to D^588^ and R^717^ in bovine ROS-GC1. The signal from GCAP1 creates a cascade of successive structural changes in the modular domains of the guanylate cyclase: the M^445^-L^450^ transduction site in the JMD (Lange et al., 1999), L^770^-P^807^ in the SHD, (Ramamurthy et al., 2001; Duda et al., 2012), and P^808^-P^964^ in the CCD (Venkataraman et al., 2008), where it is translated into the generation of cyclic GMP. The ^657^WTAPELL^663^ micro-domain flanking the N-terminal side of the SHD plays a critical role in the Ca^2+^-modulated transmission of the GCAP1 and GCAP2 signals (Figure 1B; Duda et al., 2011). Significantly, it is involved only in controlling the GCAPs-regulated activity of ROS-GC1 and does not influence the basal catalytic activity of the guanylate cyclase. Out of the seven residues constituting the motif, W^657^ controls ~70% of the total regulatory activity, due to its aromatic character. The micro-domain constitutes a hinge region of the cyclases that may strengthen dimerization interactions. The same structural motif is present in the corresponding domain of the hormone receptor ANF-RGC (Duda et al., 2009) where it controls the ATP-modulated ANF-dependent and the Ca^2+^-dependent ANF-RGC catalytic activities. We propose that this motif, embedded in a given guanylate cyclase, ties the central ligand-dependent catalytic activity to the physiological function of that cyclase, i.e., Ca^2+^ signaling to phototransduction for ROS-GC and the ANF and Ca^2+^ signaling to control of blood pressure for ANF-RGC.

The view that the L^770^-P^807^ module defines the dimeric state of the CCD and controls the basal catalytic activity (Wilson and Chinkers, 1995) has been revised. The segment consists of a five-heptad repeat that folds into a coiled coil secondary structure. Although some coiled coils actuate dimerization, this one was proposed to form a helical bridge that functionally connects the JMD, where GCAP1 binds, and the KHD to the CCD (Anantharaman et al., 2006). Accordingly, it was renamed from dimerization domain to SHD. Analysis of SHD function by mutagenesis of recombinant ROS-GC1 demonstrates that it mediates GCAP1- and GCAP2-stimulated activities (Zägel et al., 2013), however, removal of this domain completely eliminates GCAP1 signaling to the CCD without affecting regulation by GCAP2 (Zägel et al., 2013) even though both GCAPs remain bound. With the added evidence that the CCD in its isolated form exists in dimeric form and exhibits full basal catalytic activity (Venkataraman et al., 2008), the conclusion is that the SHD in ROS-GC1 selectively controls migration of the GCAP1 signal (Venkataraman et al., 2008). A prediction is that the SHD is not part of the S100B and bicarbonate pathways.

In contrast to GCAP1, the GCAP2-modulated Ca^2+^ signal originates from Y^965^-N^981^ on the C-terminal side of the CCD (Duda et al., 2005), yet it is translated at P^808^-P^964^ of the CCD, a common translational site for all signals. The protein dynamics upon Ca^2+^ decline differ for the two GCAPs (Robin et al., 2015) and unlike GCAP1, GCAP2 does not require myristoylation for ROS-GC stimulation. The remarkable 3D-structural design of CCD allows it to receive and translate these divergent non-overlapping signal pathways and translate them in independent fashions.

S100B binds to a segment within the C-terminal extension that impinges on the binding site for GCAP2. The magnitudes of stimulation by S100B and GCAP2 and their augmentation by bicarbonate were comparable (Figures 4A,C, 5A,B) suggesting that the signaling pathways for S100B and GCAP2 to the CCD may overlap.

The ROS-GC1-F^514^S mutation that is clinically linked with LCA type 1 (Perrault et al., 1996, 1999; Boye, 2015) results in a tenfold loss in ROS-GC basal catalytic activity (Duda et al., 1999). It is postulated that the loss traces to one or more of the CCD residues involved in the transformation of GTP to cyclic GMP (Figure 1B, blue-gray). Formerly, the residual 10% of the catalytic activity was thought to be insensitive to regulation by GCAP1 (Duda et al., 1999). The defect was proposed to stem from a failure of GCAP1 to interact with ROS-GC1, because the mutation locates within the GCAP1 binding site (Lange et al., 1999). However, detection of weak stimulation of F^514^S ROS-GC1 in the present study revealed that binding was preserved with an affinity that was difficult to resolve, but that appeared to fall within the normal range (Figures 3A,B). Instead of participating in binding, F^514^ may be part of the initial switch in the GCAP1 signaling pathway. The F^514^S mutant was therefore very useful as a tool in seeking deeper insight into the mechanisms by which the Ca^2+^-dependent pathways operate.

Drawing upon 3D-structurally based protein-homology (Liu et al., 1997; Venkataraman et al., 2008) and crystallographic-based models in eukaryotic Cyg12 (Winger et al., 2008), we propose that the damage to the CCD is a result of a shift in the position(s) of one (or more) GTP-specific catalytic residues (Figure 1B): D^834^, E^874^, D^878^, R^923^, C^946^, N^953^ that reside, respectively, within the following structural folds of the CCD: β1; β2; between β2 and β3; β4; between β5 and β6; and α7 (Venkataraman et al., 2008). The existence of F^514^ within the binding site for GCAP1 (Figure 1B), makes it plausible that the reduction in WT ROS-GC activity with GCAP1 at high Ca^2+^ partially invokes the same pathway as the more devastating loss in activity brought about by the mutation. Bicarbonate repaired much of the damage to basal activity of the F^514^S mutant, restoring it to nearly 70% that of normal near the end of the pathway, within the CCD. Yet bicarbonate was unable to boost GCAP1 stimulated activity at low Ca^2+^, because the intramolecular signal pathway between GCAP1 binding and catalytic activity was disrupted at an earlier stage.

The F^514^S mutation did not abrogate the GCAP2-modulated nor the S100B-modulated Ca^2+^ signaling ROS-GC1 pathway(s), although the absolute activities were reduced in all conditions. At low Ca^2+^, GCAP2 stimulated activity for a full range of 9-fold modulation, similar to the 6-fold range in the WT ROS-GC. S100B stimulated both mutant and WT ROS-GCs at high Ca^2+^ by 4–5-fold. Bicarbonate exerted a dramatic effect on GCAP2- and S100B-elevated activities of the F^514^S mutant. Stimulation was 3–4-fold greater than for WT ROS-GC, bringing the total absolute activities into closer accord. The inability of GCAP2 at high Ca^2+^ to suppress F^514^S ROS-GC1 activity may have come about because the mutant already existed in a state of maximal inhibition. These findings provide further evidence that the S100B- and GCAP2-modulated pathways overlap but that both are distinct from the GCAP1-modulated pathway. The three pathways converge with that for bicarbonate at the CCD where the bicarbonate pathway is able in large part, to overcome inhibition imposed by the F^514^S mutation under all conditions excluding co-modulation by GCAP1.

This study unfolds a new feature of phototransduction wherein the photoreceptor ROS-GC is wired as a novel sensor of bicarbonate, allowing for potent, Ca^2+^-independent enhancement of cyclic GMP synthesis. It mechanistically demonstrates that the bicarbonate signaling pathway is interlinked synergistically with Ca^2+^-dependent pathways in regulating ROS-GC in rod and cone phototransduction. The mechanisms underlying the GCAP linkages differ; at the most basic levels the two GCAPs-modulated pathways do not overlap. On the other hand, S100B may tap into the GCAP2 pathway. Since most, if not all, ROS-GC molecules exist as a stable complex with GCAP or S100B in photoreceptors, the physiological role of bicarbonate is to amplify the Ca^2+^-dependent switching behavior of ROS-GC by 2-fold or more. The F→S mutation in ROS-GC1 responsible for LCA1 dystrophy substantially reduces the rate of cyclic GMP synthesis and damages specifically, modulation by GCAP1. Bicarbonate has the capacity to restore activity to nearly normal levels with GCAP2 or with S100B bound under conditions of low Ca^2+^, but was incapable of reinstating full modulation by GCAP1. Cones selectively express GCAP1, thus bicarbonate cannot alleviate the deficiency in cyclic GMP synthesis required for photopic vision in LCA1 patients. However, if bicarbonate were to reach high enough levels in rods, it might support adequate cyclic GMP synthesis because they express GCAP2 as well as GCAP1. There are other heritable, degenerative retinal diseases caused by dysregulation of cyclic GMP synthesis or by hypersensitivity of the CNG channel to cyclic GMP. As a powerful modulator of ROS-GC, bicarbonate is likely to influence the penetrance and severity of these diseases. Control over bicarbonate production could have therapeutic benefits. Finally, CO_2_ may constitute a common signaling theme of the photoreceptor and the odorant membrane guanylate cyclases.

## Author Contributions

TD designed, carried out and analyzed the experiments. AP expressed and purified GCAPs, and isolated outer segment membranes from mice. CLM helped plan the experiments, critically analyzed and interpreted the results, and prepared the figures. RKS conceptually planned and coordinated the study and generated the model. All authors contributed to the writing of the manuscript.

## Conflict of Interest Statement

The authors declare that the research was conducted in the absence of any commercial or financial relationships that could be construed as a potential conflict of interest.

## Footnotes

^1^http://web.stanford.edu/~cpatton/CaMgATPEGTA-NIST.htm

## Abbreviations

ANF-RGC: atrial natriuretic factor (ANF) receptor guanylate cyclase
CCD: core catalytic domain
CNG: cyclic nucleotide gated
Cyg12: guanylate cyclase from green algae *Chlamydomonas reinhardtii*
GCAP: guanylate cyclase activating protein
JMD: juxtamembrane domain
KHD: kinase homology domain
LCA: Leber’s congenital amaurosis
PDE: phosphodiesterase
ROS-GC: rod outer segment guanylate cyclase
SHD: signaling helix domain.
